# A dosimetric evaluation of VMAT for the treatment of non‐small cell lung cancer

**DOI:** 10.1120/jacmp.v14i1.4110

**Published:** 2012-09-01

**Authors:** Caitlin E. Merrow, Iris Z. Wang, Matthew B. Podgorsak

**Affiliations:** ^1^ Department of Physiology and Biophysics State University of New York at Buffalo, SUNY at Buffalo, and Department of Radiation Medicine, Roswell Park Cancer Institute Buffalo NY USA

**Keywords:** volumetric‐modulated arc therapy (VMAT), stereotactic body radiation therapy (SBRT), three‐dimensional conformal radiation therapy (3D CRT), non‐small cell lung cancer (NSCLC)

## Abstract

The purpose of this study was to demonstrate the dosimetric potential of volumetric‐modulated arc therapy (VMAT) for the treatment of patients with medically inoperable stage I/II non‐small cell lung cancer (NSCLC) with stereotactic body radiation therapy (SBRT). Fourteen patients treated with 3D CRT with varying tumor locations, tumor sizes, and dose fractionation schemes were chosen for study. The prescription doses were 48 Gy in 4 fractions, 52.5 Gy in 5 fractions, 57.5 Gy in 5 fractions, and 60 Gy in 3 fractions for 2, 5, 1, and 6 patients, respectively. VMAT treatment plans with a mix of two to three full and partial noncoplanar arcs with 5°–25° separations were retrospectively generated using Eclipse version 10.0. The 3D CRT and VMAT plans were then evaluated by comparing their target dose, critical structure dose, high dose spillage, and low dose spillage as defined according to RTOG 0813 and RTOG 0236 protocols. In the most dosimetrically improved case, VMAT was able to decrease the dose from 17.35 Gy to 1.54 Gy to the heart. The $D_{2\text{cm}}$ decreased in 11 of 14 cases when using VMAT. The three that worsened were still within the acceptance criteria. Of the 14 3D CRT plans, seven had a $D_{2\text{cm}}$ minor deviation, while only one of the 14 VMAT plans had a $D_{2\text{cm}}$ minor deviation. The $R_{50\%}$ improved in 13 of the 14 VMAT cases. The one case that worsened was still within the acceptance criteria of the RTOG protocol. Of the 14 3D CRT plans, seven had an $R_{50\%}$ deviation. Only one of the 14 VMAT plans had an $R_{50\%}$ deviation, but it was still improved compared to the 3D CRT plan. In this cohort of patients, no evident dosimetric compromises resulted from planning SBRT treatments with VMAT relative to the 3D CRT treatment plans actually used in their treatment.

PACS numbers: 87.50.‐a, 87.53.‐j, 87.55.‐x, 87.55.D‐, 87.55.dk, 87.55.de

## I. INTRODUCTION

Non‐small cell lung cancer (NSCLC) accounts for approximately 75%–80% of all patients diagnosed with lung carcinoma. Of the NSCLC patients, only 10%–15% have early stage, localized no nodal involvement. The preferred treatment of such patients is surgical resection; however, many patients are deemed medically inoperable due to physical limitations that could result in an inadequate surgery or recovery period.^(^ ^1^ ^)^

Stereotactic body radiation therapy (SBRT) is a form of radiation therapy that delivers high levels of radiation dose of 10–30 Gy per fraction.^(^ ^2^ ^–^ ^15^ ^)^ This hypofractionation technique is used in conjunction with respiratory motion management, four‐dimensional computed tomography (4D CT), and image guidance to ensure accuracy.^(^ ^2^ ^)^ This treatment technique has been shown to have better results for local recurrence when compared to nonanatomic wedge resections.^(^ ^3^ ^)^ With three‐year actuarial local control rates of 98% and survival rates of 58%, SBRT gives surgically inoperable patients a comparable treatment option for tumor control.^(^ ^7^ ^)^

Conformal dose distributions and steep dose gradients are essential when treating SBRT and are achieved by: (a) having little to no margins around the target volume to account for penumbra, (b) using an abundant amount of coplanar or noncoplanar fields, and (c) using inhomogeneous dose distributions within the target volume.^(^ ^4^ ^,^ ^5^ ^)^

Three‐dimensional conformal radiation therapy (3D CRT) is the most common form of treatment planning for SBRT. Anywhere from 10–15 static fields are used to create the desired conformal dose distribution. The main drawback to this approach is the lengthy treatment times that occur as a result of the many fields needed to create an acceptable treatment plan.^(^ ^4^ ^)^ RTOG 0236 showed that only 75% of patients treated with this approach avoided severe toxicity, the remaining 25% did not.^(^ ^7^ ^)^ There have been several efforts to improve upon the avoidance of severe toxicity and to decrease the dose to healthy tissue for SBRT lung cases. These efforts included techniques such as intensity‐modulated radiation therapy (IMRT) and conformal arc therapy.^(^ ^8^ ^–^ ^10^ ^)^

An alternative method of treatment planning that is becoming a more attractive option for the delivery of SBRT is volumetric‐modulated arc therapy (VMAT), also known as RapidArc. VMAT delivers dose to the target volume in a full 358° rotation with varying gantry speed, multileaf collimator (MLC) positions, and dose rate.^(^ ^2^ ^)^ These capabilities allow VMAT to increase the sparing of organs at risk without compromising conformal dose distributions, while significantly reducing treatment time.

It was hypothesized that the use of multiple noncoplanar arcs as opposed to multiple noncoplanar static gantry fields could potentially decrease the maximum dose delivered to organs at risk and, in turn, increase the percentage of patients able to avoid severe toxicity, while increasing the conformality of the dose to the target volumes.

The purpose of this study was to demonstrate the dosimetric potential of volumetric‐modulated arc therapy (VMAT) for the treatment of patients with medically inoperable stage I/II non‐small cell lung cancer (NSCLC) with stereotactic body radiation therapy (SBRT).

## II. MATERIALS AND METHODS

### A. Patient selection

Fourteen previously treated patients with medically inoperable stage I/II NSCLC were chosen (Table 1). The cohort of patients was selected in order to study various tumor locations within the lung, tumor sizes, and dose fractionation schemes. All 14 patients were evaluated using RTOG protocol parameters. Of the 14 cases, three had a tumor located in the right upper lobe (RUL), two in the right middle lobe (RML), two in the right lower lobe (RLL), five in the left upper lobe (LUL), and two in the left lower lobe (LLL).

**Table 1:** Patient characteristics.

*Case #*	*PTV (cc)*	*GTV (cc)*	*Tumor Location*	*Age (yr)*	*Gender*
1	75.53	35.41	RUL	75	Male
2	21.39	5.70	LUL	79	Male
3	41.35	10.22	RUL	66	Female
4	23.73	4.77	RUL	77	Male
5	31.00	6.73	LUL	82	Female
6	22.73	4.24	LUL	81	Male
7	40.00	9.71	LUL	90	Male
8	18.03	4.46	LUL	77	Female
9	26.54	9.37	RML	70	Male
10	31.26	7.50	RML	85	Female
11	25.52	5.57	RLL	83	Male
12	34.10	8.54	RLL	75	Male
13	63.45	18.45	LLL	88	Female
14	18.26	5.01	LLL	78	Female

### B. Treatment planning volumes

4D CTs with a 1.25 mm slice thickness were obtained using Varian's real‐time positioning management (RPM) system (Varian Medical Systems, Palo Alto, CA) around the region of interest. This RPM system uses an infrared block placed on the patient's chest during data acquisition for breathing phase and motion tracking information. Abdominal compression plates were used during data acquisition and during treatment to restrict the motion of the tumor due to respiration.^(^ ^12^ ^)^ Boolean operators were used to obtain the tumor motion‐encompassing target volume (ITV). Extended CT slices were acquired every 1.25 mm for critical structure acquisition.

The gross tumor volume (GTV) and the clinical tumor volume (CTV) were equivalent for all plans. The planning target volume (PTV) was defined as the GTV (equivalent to the ITV described above) increased 0.5 cm in the axial plane and 1.0 cm in the cranial–caudal plane. The PTVs of the 14 cases ranged from $18.03-75.53\;{\text{cm}}^{3}$, with GTVs ranging from $4.46-35.41\;{\text{cm}}^{3}$. Contouring of normal structures strictly followed RTOG 0236 and RTOG 0813 protocol standards, with the exception of the additional contouring of the single right lung and the single left lung. This was done to provide dosimetric information of the two separate lungs in addition to the information of the two lungs as a whole. Structure sets from the original 3D CRT treatment plans were utilized during VMAT treatment planning in order to eliminate uncertainties due to differences in contouring.

### C. Dose‐volume constraints

The target prescription doses were 48 Gy in 4 fractions, 52.5 Gy in 5 fractions, 57.5 Gy in 5 fractions, and 60 Gy in 3 fractions for two, five, one, and six patients, respectively. The two patients with slightly modified fractionation schemes of 48 Gy in 4 fractions were still evaluated using dose constraints of the previously mentioned RTOG protocols.

The prescription line for PTV coverage was to be above 60% and below 90%, according to the RTOG protocols. This results in an increase in hot spots that are to be manipulated to occur within the tumor volume. These hot spots are equal to the reciprocal of the prescription line used. The protocols requires 95% of the PTV to receive 100% of the prescribed dose, while 99% of the PTV is to receive 90% of the prescribed dose.^(^ ^1^ ^,^ ^7^ ^)^ The conformality index is the ratio of the total volume that receives 100% of the prescribed dose to the PTV and was to be ≤1.2, with a minor deviation of 1.5.

The amount of normal tissue that receives 105% of the prescription dose should not exceed 15% of the PTV volume. The maximum dose to normal tissue 2.0 cm in all directions from the PTV, ($D_{2\text{cm}}$), was not to exceed 50%–77% of the prescribed dose (57%–94% for minor deviations) for patients following RTOG 0813, and 28.1–44.3 Gy (30.1–46.3 Gy for minor deviations) for those following RTOG 0236. The ratio of the volume that receives 50% of the prescribed dose to the PTV, ($R_{50\%}$), was not to exceed 2.9–5.9 (3.7–7.5 for minor deviations) for patients following RTOG 0813, and 2.9–3.9 (3.1–4.1 for minor deviations) for those following RTOG 0236. These ranges correlate to the varying sizes of the tumor volumes and are depicted in the RTOG protocols.^(^ ^1^ ^,^ ^6^ ^)^ High dose spillage, $D_{2\text{cm}}$, and $R_{50\%}$ constraint variations depend on size of PTV.

For the RTOG 0813 patients, the volume of each lung receiving 12.5 Gy and 13.5 Gy, ($V_{12.5}$ and $V_{13.5}$), was to be below $1500\;{\text{cm}}^{3}$ and $1000{\text{cm}}^{3}$, respectively. For all patients, the volume of the total lung that receives 20 Gy, ($V_{20}$), was to be kept below 10% (15% for minor deviations).

In order to avoid radiation toxicity to healthy organs at risk (OARs), maximum point dose limitations that have been suggested in the RTOG protocols were followed (Tables 2 and 3).

**Table 2:** Maximum dose point to the organs at risk (OARs) of the eight RTOG 0813 cases.

*Critical Structures*	*RTOG 0813: Max Dose to Any Point (Gy)*	*Max Dose Point 3DCRT (Gy) Mean(Range)*	*Max Dose Point VMAT (Gy) Mean(Range)*
Spine	30.0	15.91 (4.43–28.44)	11.15 (3.34–27.59)
Brachial Plexus	32.0	12.42 (0.06–42.22)	9.87 (0.03–31.93)
Skin	32.0	19.62 (12.45–27.86)	13.30 (7.92–21.17)
Esophagus	50.4–60.4	19.09 (5.40–54.47)	15.58 (3.36–52.05)
Great Vessels	50.4–60.4	24.60 (9.55–42.92)	22.25 (8.33–40.62)
Trachea	50.4–60.4	13.30 (0.11–49.86)	11.16 (0.07–41.25)
Proximal Bronchial Tree	50.4–60.4	15.47 (3.09–53.62)	11.78 (1.95–42.85)
Heart	50.4–60.4	14.04 (3.96–31.05)	10.50 (1.54–32.42)

**Table 3:** Maximum dose point to the organs at risk (OARs) of the six RTOG 0236 cases.

*Critical Structures*	*RTOG 0236: Max Dose to Any Point (Gy)*	*Max Dose Point 3DCRT (Gy) Mean(Range)*	*Max Dose Point VMAT (Gy) Mean(Range)*
Spine	18.0	10.88 (3.11–17.31)	6.90 (2.77–11.92)
Brachial Plexus	24.0	2.28 (0.10–8.47)	2.18 (0.16–7.64)
Esophagus	27.0	13.59 (7.97–26.67)	8.87 (5.37–16.52)
Trachea	30.0	7.92 (0.22–27.94)	5.36 (0.20–11.74)
Proximal Bronchial Tree	30.0	24.51 (2.35–58.16)	22.56 (2.66–54.47)
Heart	30.0	22.51 (2.60–42.8)	18.60 (2.43–36.02)

### D. Treatment planning

All 3D CRT plans were planned using Varian's Eclipse treatment planning system (TPS) version 8.5 (Varian Medical Systems). All VMAT plans were planned using Varian's Eclipse version 10.0. Version 10.0's capability of optimizing multiple noncoplanar arcs was more advanced and computationally less time‐consuming than a multiple noncoplanar arc treatment plan on version 8.5. Isocenter was chosen to correspond closely to the center of mass of the PTV. The normalization point was chosen to be the isocenter. Tissue heterogeneity corrections were applied during dose calculations to 10 of the VMAT treatment plans and 10 of the 3D CRT treatment plans. The remaining four VMAT and 3D CRT plans were not calculated using tissue heterogeneity corrections. This was due to RTOG 0236 protocol requirements stating tissue heterogeneity corrections should not be used for the purpose of dose planning and calculation of monitor units for actual treatment. Since the four previously treated 3D CRT treatment plans followed protocol and did not use heterogeneity corrections, the newly generated corresponding VMAT plans also did not use tissue heterogeneity corrections, in order to eliminate heterogeneity corrections as a variable in the results. All plans used 6 MV photons. The 3D CRT plans used 10–13 noncoplanar fields, while the VMAT plans utilized three partial and/or full noncoplanar arcs with a 5°–25° separation. Collimator angles of 45°, 325°, and 30° were chosen in the VMAT plans to minimize MLC tongue‐and‐groove effects.

At the beginning of optimization, all of the OARs were given a priority of 90. A structure equal to the PTV+3 mm in the superior and inferior directions called PTVx for all cases was made and used during optimization; this was to aid the conformality and coverage of the PTV. Upper and lower objectives with priorities of 150 and 130, respectively, were used for the GTV, PTV, and PTVx for all of the cases. For the GTV, PTV, and PTVx, the upper objectives were 0.0% of the volume receives 105% of the prescription dose, and the lower objectives were 100.0% of the volume receives 100% of the prescription dose for all cases. Optimization was performed twice for every case. During the first optimization, no priorities or objectives were changed. After the first optimization cycle, a structure was made from the 50% dose line. The 50% dose structure was used during the second optimization process in order to decrease the $R_{50\%}$. All doses to the OARs were first set to the RTOG recommended dose limits, but were changed to more suitable doses once the first level of optimization was complete. The priorities of the OARs were increased throughout the optimization levels to further decrease the dose to these critical structures without compromising the PTV coverage.

The PTVs were given the highest priorities (150 upper and 130 lower). Generally, the lung structures and 50% dose structures were given the second highest priorities (115–120, all upper limits). All other structures had varying priorities of 90–110 (all upper limits). Due to variations of patient anatomy and tumor locations, it is difficult to give a definitive explanation as to which structures were given high priorities and which were not.

### E. Evaluation of treatment plans

The 3D CRT and VMAT plans were evaluated by comparing their target dose, critical structure dose, high dose spillage, and low dose spillage as defined according to RTOG 0813 and RTOG 0236 protocols. $V_{20}$, $V_{13.5}$, $V_{12.5}$, and $V_{5}$ values were recorded and compared for both right and left lung volumes for the patients following RTOG 0813. Only $V_{20}$ and $V_{5}$ values for both right and left lung volumes were recorded and compared for patients following RTOG 0236. The conformality index (CI), $D_{2\text{cm}}$, $R_{50\%}$, as well as normal tissue minus PTV values, were recorded and compared for all 14 3D CRT and VMAT treatment plans.

### F. Data analysis

All 3D CRT plans were compared to VMAT plans. The 14 patients were grouped into those evaluated using RTOG 0813 (eight patients) and those evaluated using RTOG 0236 (six patients) acceptance criteria (Table 4). Dose to critical structures, $V_{20}$, $V_{13.5}$, $V_{12.5}$, and $V_{5}$, conformality ratios, and normal tissue values were compared by averaging for each of the two groups (Tables 5 and 6).

**Table 4:** RTOG protocols and total monitor units (MUs).

*Case #*	*RTOG Protocol*	*Prescription*	*Total MUs 3D CRT*	*Total MUs VMAT*
1	0813	52.5 Gy in 5 Fractions	1583	3543
2	0813	52.5 Gy in 5 Fractions	2215	4891
3	0813	48 Gy in 4 Fractions	2926	5198
4	0813	52.5 Gy in 5 Fractions	1933	4583
5	0813	52.5 Gy in 5 Fractions	3051	4613
6	0813	48 Gy in 4 Fractions	2425	5439
7	0813	52.5 Gy in 5 Fractions	1822	4144
8	0813	57.5 Gy in 5 Fractions	4519	5629
9	0236	60 Gy in 3 Fractions	4894	8310
10	0236	60 Gy in 3 Fractions	4396	7212
11	0236	60 Gy in 3 Fractions	3419	6731
12	0236	60 Gy in 3 Fractions	6135	8539
13	0236	60 Gy in 3 Fractions	5531	6533
14	0236	60 Gy in 3 Fractions	6903	7969

**Table 5:** Dose conformality and dose spillage results of the eight RTOG 0813 cases.

*Conformity/Dose Spillage Index*	*RTOG 0813 Limits*	*3D CRT Mean(Range)*	*VMAT Mean(Range)*
$V_{20}$‐Right Lung (%)	≤10% (up to 15%)	1.44% (0.00–5.67%)	1.31% (0.00–5.75%)
$V_{20}$‐Left Lung (%)	≤10% (up to 15%)	6.22% (0.00–12.35%)	5.24% (0.00–11.22%)
$V_{5}$‐Right Lung (%)		7.61% (0.68–29.36%)	7.53% (0.29–28.56%)
$V_{5}$‐Left Lung (%)		18.86% (0.00–41.96%)	17.43% (0.14–37.71%)
$V_{12.5}$‐Left Lung (cc)	≤ 1500 cc of the lung can receive 12.5 Gy	143.57 cc (0.00–283.39 cc)	121.13 cc (0.00–250.11 cc)
$V_{12.5}$‐Right Lung (cc)		99.69 cc (0.00–446.22 cc)	77.49 cc (0.00–302.58 cc)
$V_{13.5}$‐Left Lung (cc)	≤ 1000 cc of the lung can receive 13.5 Gy	133.21 cc (0.00–260.55 cc)	111.99 cc (0.00–233.88 cc)
$V_{13.5}$‐Right Lung (cc)		90.95 cc (0.00–418.01 cc)	69.52 cc (0.00–284.21 cc)
Conformality Index	≤1.2 (up to 1.5)	1.17 (1.05–1.35)	1.03 (1.00–1.15)
NT (%)	≤(15%${\text{xPTV}}_{\text{vol}}$) can receive 105% of the Prescribed Dose	8.19% (0.18–27.56%)	1.82% (0.00–10.17%)

Note: $V_{20}$ is the volume receiving 20 Gy, $V_{12.5}$ is the volume receiving 12.5 Gy, $V_{13.5}$ is the volume receiving 13.5 Gy $V_{5}$ is the volume receiving 5 Gy, and NT is the normal tissue receiving 105% of the prescribed dose.

**Table 6:** Dose conformality and dose spillage results of the six RTOG 0236 cases.

*Conformity/Dose Spillage Index*	*RTOG 0236 Limits*	*3D CRT Mean(Range)*	*VMAT Mean(Range)*
$V_{20}$‐Right Lung (%)	≤10% (up to 15%)	7.36% (0.13–12.35%)	5.57% (0.00–11.84%)
$V_{20}$‐Left Lung (%)	≤10% (up to 15%)	2.33% (0.00–12.35%)	1.81% (0.00–10.82%)
$V_{5}$‐Right Lung (%)		23.82% (7.83–39.51%)	24.95% (1.49–47.00%)
$V_{5}$‐Left Lung (%)		7.40% (0.00–26.17%)	6.39% (0.00–28.23%)
Conformality Index	≤.2 (up to 1.5)	1.15 (1.06–1.22)	1.006 (1.00–1.01)
NT(%)	≤(15%${\text{xPTV}}_{\text{vol}}$) can receive 105% of the Prescribed Dose	8.26% (2.78–15.02%)	0.31% (0.06–0.91%)

Note: $V_{20}$ is the volume receiving 20 Gy, $V_{5}$ is the volume receiving 5 Gy and NT is the amount of normal tissue receiving 105% of the prescribed dose.

## III. RESULTS & DISCUSSION

The VMAT treatment plans yielded on average a 9.6%–33.7% reduction in dose to critical structures and an average 12.0%–12.5% increase in conformality compared with 3D CRT. These results are consistent with Zhang et al.^(^ ^16^ ^)^ where coplanar and noncoplanar VMAT plans, with and without a flattening filter, were created and compared to 3D CRT treatment plans for 15 SBRT lung cases. The amount of normal tissue receiving 105% of the prescription dose decreased on average 6.30%–7.95%. The overall dosimetrically best case reduced the dose to the heart from 17.35 Gy in the 3D CRT plan to 1.54 Gy in the VMAT plan. The greatest increase of dose to any given critical structure for all of the 14 plans was a dose of 4.06 Gy to the heart in the 3D CRT plan increased to 7.53 Gy to the heart in the VMAT plan, giving an overall greatest increase in absolute dose of only 3.47 Gy. Although it was not desired, the increase of dose to the heart was still well under the maximum dose point limit of 55.1 Gy — 105% of 52.5 Gy in this case — of the RTOG 0813 protocol.

Zhang et al.^(^ ^16^ ^)^ achieved slightly better results regarding dose homogeneity in the target volumes when using the flattening filter free VMAT technique and with reducing the contralateral lung dose by choosing partial arcs to avoid the contralateral lung. McGrath et al.^(^ ^17^ ^)^ used dose regimens of 48 Gy in 12 fractions, but also chose partial arcs to avoid the contralateral lung and achieved similar end results. Our study did not do this and instead generally chose one full arc and two partial arcs to yield noncoplanar arcs separated by 10°–25°. The range of the arcs was determined by mechanical collision limitations and not in order to avoid any critical structures. If gantry and couch collisions limited the range of the arc a smaller degree of separation, 5°, between arcs was chosen. It is important to point out that this study was still able to produce an increase in dose conformality to the target volumes and, on average, decrease dose to the contralateral lung, even with this method of choosing arc angles and degrees of rotation.

**Figure 1:**
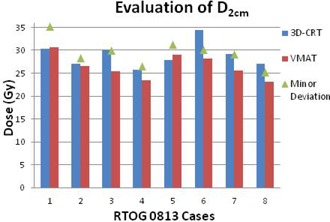
Evaluation of the maximum dose point 2 cm from the planning target volume in any direction for the eight patients evaluated using RTOG 0813 protocol criteria.

**Figure 2:**
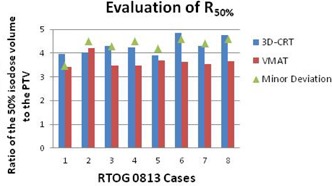
Evaluation of the ratio of the 50% isodose volume to the planning target volume for the eight patients evaluated using RTOG 0813 protocol criteria. Cases 1, 2, 3, 4, 5, 6, 7, and 8 had PTVs of $75.53\;{\text{cm}}^{3}$, $21.39\;{\text{cm}}^{3}$, $31.00\;{\text{cm}}^{3}$, $22.73\;{\text{cm}}^{3}$, $40.00\;{\text{cm}}^{3}$, $18.03\;{\text{cm}}^{3}$, $26.54\;{\text{cm}}^{3}$, and $18.26\;{\text{cm}}^{3}$, respectively.

**Figure 3:**
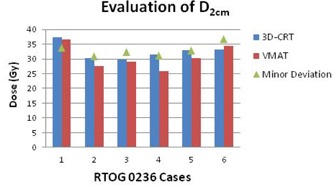
Evaluation of the maximum dose point 2 cm from the planning target volume in any direction for the six patients evaluated using RTOG 0236 protocol criteria.

**Figure 4:**
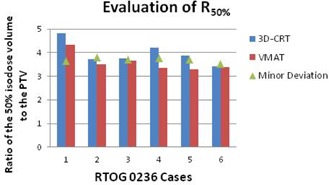
Evaluation of the ratio of the 50% isodose volume to the planning target volume for the six patients evaluated using RTOG 0236 protocol criteria. Cases 1, 2, 3, 4, 5, and 6 had PTVs of $41.35\;{\text{cm}}^{3}$, $23.73\;{\text{cm}}^{3}$, $31.26\;{\text{cm}}^{3}$, $25.52\;{\text{cm}}^{3}$, $34.10\;{\text{cm}}^{3}$, and $63.45\;{\text{cm}}^{3}$, respectively.

The $D_{2\text{cm}}$ improved with VMAT in 11 of 14 cases. The three that worsened were still within the acceptance criteria. Of the 14 3D CRT plans, seven had a $D_{2\text{cm}}$ deviation. Only one of the 14 VMAT plans had a $D_{2\text{cm}}$ deviation and, in this case, still improved compared to the original 3D CRT plan. The $R_{50\%}$ improved in 13 of the 14 cases. The one case that worsened was still within the acceptance criteria. Of the 14 3D CRT plans, seven had an $R_{50\%}$ deviation. Only one of the 14 VMAT plans had an $R_{50\%}$ deviation, but was still dosimetrically superior to the originally treated 3D CRT plan (Figures 1–4).

After comparing the two different treatment planning techniques using the RTOG 0813 and 0236 protocols as a comparison guide, VMAT became dosimetrically more promising.

It was noted that the size of the tumor did not hinder the improved capabilities of VMAT to deliver a dosimetrically improved plan. Regardless of the tumor size, it was still possible to decrease the dose to critical structures, increase conformality of the dose to the target volume, and limit the dose spillage to healthy tissue using VMAT in comparison to the 3D‐CRT technique. Note that the tumor sizes were the sizes indicated to be allowed by the RTOG protocols. In addition to tumor size, tumor location and dose fractionation schemes were also studied and noted to have an insignificant impact on the results.

It was found that with increasing the degree of separation between arcs, the $R_{50\%}$, $D_{2\text{cm}}$, $V_{20}$, $V_{12.5}$, $V_{13.5}$, $V_{5}$, and dose to the skin would decrease. It was also found that producing a dosimetrically improved plan with ≤5° separation between arcs was possible yet difficult, and that a separation of at least 10° was preferred and a separation of 25° between arcs and/or partial arcs was ideal. Most plans were allowed one full 360° arc at a couch position of 0° complimented by two partial arcs at couch positions of 335°–350° and 10°–25°. One case required three partial arcs due to extreme vertical, longitudinal, and lateral couch positions that caused gantry‐couch collisions even with a couch position of 0°. Appropriate collision checks were preformed and all of the plans were verified to be deliverable. This method of choosing arc angles and positions may be the reason why a small increase of dose to critical structures was seen in a small number of the plans.

Even with the use of three full and/or partial arcs, the treatment time is still expected to be significantly reduced in comparison to 10–13 static fields used in traditional 3D CRT. While there may be reductions in treatment delivery time, the treatment planning time could increase due to the extra needed time for optimization, trial and error approaches when attempting to meet dose constraints, and extra needed time for contouring the extended CT data that is essential for properly optimizing VMAT. This is consistent with Ong et al.^(^ ^9^ ^)^ and Brock et al.,^(^ ^10^ ^)^ where both papers mentioned treatment planning times for VMAT of 1–3 hours when using Eclipse version 8.2. The Brock study reported an increase in treatment planning time even with the utilization of breath‐hold techniques that eliminate the extra time needed for additional contouring of the tumor motion‐encompassing volume that was used in this study and in Ong et al.^(^ ^9^ ^)^ Due to the ability of Eclipse version 10.0 to optimize multiple arcs at once, the treatment planning times are slightly decreased when using the latest TPS version but still increased in comparison to 3D CRT treatment planning.

In another study comparing 3D CRT to VMAT for the treatment of lung cancer,^(^ ^18^ ^)^ only one full or partial arc was used to deliver the radiation dose to the target volumes of 12 patients. They were able to decrease the $V_{20}$ when using VMAT compared to 3D CRT. This ability to deliver the radiation dose in only one full or partial arc and meet clinically acceptable standards was due to the decrease in the dose regimen, 68 Gy in 34 fractions, compared to this study where the dose regimens are about 50 Gy in 5 fractions and 60 Gy in 3 fractions. Due to the increase in dose per fraction in this study, it was important to use multiple arcs. This is consistent with Verbakel et al.^(^ ^19^ ^)^ who used at least two arcs to deliver hypofractionated doses to lung tumors.

The RTOG protocols state that no field size less than $3.5\;{\text{cm}}^{2}$ should be used during treatment planning. This is due to the wider range of the secondary Compton electrons and the loss of charged particle equilibrium (CPE) that occurs at the interfaces of vastly different electron densities that become increasingly pronounced with decreasing field sizes. The current ability of dose algorithms to account for such occurrences is lacking, and leads to discrepancies between calculated dose and actual dose delivered.^(^ ^16^ ^)^ It is well known that Monte Carlo simulations are most accurate when accounting for such occurrences, but unfortunately result in lengthy calculation times.

Seppala et al.^(^ ^20^ ^)^ showed that pencil beam convolution (PBC) algorithms used to calculate target dose would overestimate dose for smaller target margins (1.5 cm) and peripheral target dose for larger target margins (2–5 cm) for tumor volumes surrounded by lung tissue, resulting in an underdosing of the tumor volume when compared to the calculated dose in the treatment planning system (TPS). Anisotropic analytic algorithm (AAA) was shown to be more accurate when calculating target dose for both the smaller and larger target volumes when compared to PBC; however, underestimated the peripheral dose to the target volumes when compared to the EBT2 film measurements, resulting in a slight overdosing to the tumor volume when compared to the calculated dose in the TPS. PBC failed to meet the ± 3%/± 1 mm gamma evaluation on average 61% (yielding an average pass rate of 39%) for the smaller defined target volume, while AAA failed on average 16% (yielding an average pass rate of 84%). When using AAA, the measured 80%–95% isodose lines had an average difference of 1.1 mm when compared to the isodose lines in the TPS.^(^ ^20^ ^)^ It was found by Seppala and colleagues that a minimum aperture distance, using the sliding window aperture technique, of 6 mm should be used in order to ensure accuracy between dose delivered and dose calculated.

During this study, AAA was used to calculate dose to the target volumes in all of the VMAT treatment plans. The number of MLC pairs that had a separation greater than 6 mm was documented. On average, 80.25% of the MLC leaf gaps exceeded 6 mm. Since this number does not correlate to point of occurrence on the PTV, it cannot be concluded that this would affect a large percentage of the overall dose delivered. Further study of the VMAT fields needs to be preformed to quantify the discrepancies between dose delivered and dose calculated.

Based on the previously mentioned study,^(^ ^20^ ^)^ it can be presumed that all of the VMAT treatment plans can deliver an adequate treatment when AAA is used and when the vast majority of the MLC gaps are kept above 6 mm.

In this study, it was shown that when generating treatment plans using VMAT with dose constraints in mind, it is possible to create a clinically acceptable treatment plan and, in most cases, an improved plan when comparing to 3D CRT plans.

## IV. CONCLUSIONS

3D CRT can provide SBRT lung patients a highly conformal dose to tumor volumes and keep irradiation of healthy tissue below acceptance criteria. However, VMAT shows great potential for producing an increase in conformal dose to the tumor volumes and an increase of the sparing of organs at risk when compared to the previously mentioned treatment delivery technique.

In this study, no evident dosimetric compromises resulted from planning SBRT treatments with VMAT relative to the 3D CRT treatment plans actually used in their treatment. It was found that the VMAT planning technique has the potential to be dosimetrically superior for SBRT treatment of stage I/II NSCLC compared to traditionally used 3D CRT.
